# The Novel Alpha-2 Adrenoceptor Inhibitor Beditin Reduces Cytotoxicity and Huntingtin Aggregates in Cell Models of Huntington’s Disease

**DOI:** 10.3390/ph14030257

**Published:** 2021-03-12

**Authors:** Elisabeth Singer, Lilit Hunanyan, Magda M. Melkonyan, Jonasz J. Weber, Lusine Danielyan, Huu Phuc Nguyen

**Affiliations:** 1 Institute of Medical Genetics and Applied Genomics, University of Tuebingen, Calwerstrasse 7, 72076 Tuebingen, Germany; Elisabeth.singer@med.uni-tuebingen.de (E.S.); jonasz.weber@med.uni-tuebingen.de (J.J.W.); 2 Centre for Rare Diseases (ZSE), University of Tuebingen, Calwerstrasse 7, 72076 Tuebingen, Germany; 3 Department of Human Genetics, Ruhr University Bochum, Universitaetsstrasse 150, 44801 Bochum, Germany; 4 Department of Medical Chemistry, Yerevan State Medical University, Yerevan 0025, Armenia; h-lilit@live.com (L.H.); magda.melkonyan@meduni.am (M.M.M.); 5 Department of Clinical Pharmacology, University Hospital of Tuebingen, 72076 Tuebingen, Germany; lusine.danielyan@med.uni-tuebingen.de; 6 Department of Biochemistry and Neuroscience Laboratory, Yerevan State Medical University, Yerevan 0025, Armenia

**Keywords:** Huntington’s disease, huntingtin, neurodegeneration, alpha-2 adrenoceptor, beditin, autophagy, neuronal cell survival

## Abstract

Huntington’s disease (HD) is a monogenetic neurodegenerative disorder characterized by the accumulation of polyglutamine-expanded huntingtin (mHTT). There is currently no cure, and therefore disease-slowing remedies are sought to alleviate symptoms of the multifaceted disorder. Encouraging findings in Alzheimer’s and Parkinson’s disease on alpha-2 adrenoceptor (α2-AR) inhibition have shown neuroprotective and aggregation-reducing effects in cell and animal models. Here, we analyzed the effect of beditin, a novel α2- adrenoceptor (AR) antagonist, on cell viability and mHTT protein levels in cell models of HD using Western blot, time-resolved Foerster resonance energy transfer (TR-FRET), lactate dehydrogenase (LDH) and terminal deoxynucleotidyl transferase dUTP nick end labeling (TUNEL) cytotoxicity assays. Beditin decreases cytotoxicity, as measured by TUNEL staining and LDH release, in a neuronal progenitor cell model (ST*Hdh* cells) of HD and decreases the aggregation propensity of HTT exon 1 fragments in an overexpression model using human embryonic kidney (HEK) 293T cells. α2-AR is a promising therapeutic target for further characterization in HD models. Our data allow us to suggest beditin as a valuable candidate for the pharmaceutical manipulation of α2-AR, as it is capable of modulating neuronal cell survival and the level of mHTT.

## 1. Introduction

Huntington’s disease (HD) is a fatal, autosomal dominantly inherited neurodegenerative disorder caused by the expansion of a polyglutamine (polyQ)-coding CAG repeat within the mutant huntingtin gene [1]. Patients suffer from motoric, emotional, and cognitive dysfunctions with typical onset of disease at mid-age and approximately 20 years of disease progression after diagnosis. There is currently no treatment to slow or halt the disease. The disease is characterized by accumulations of the polyQ-expanded protein and therefore the currently most promising approach to treat HD is reducing the mutant protein by RNA interference or antisense oligonucleotides (ASOs) [2]. While protein accumulations were characterized early on as a hallmark of the disease, the pathogenic mechanisms caused by the mutant protein are less well understood. It has been established that excitotoxicity, altered transcription, and deficient degradation cause the mutant protein to display cytotoxic properties [3], leading to neurodegeneration that starts in the medium spiny neurons of the striatum and extends to other brain regions with disease progression [4].

α2-adrenergic receptors (α2-AR) are widely expressed in human and rodent brains [5]. α2-ARs are coupled to G-proteins and exert mostly inhibitory functions. In mammals, three subtypes exist: α2A–C (ADRA2-A/B/C), which signal through different G-proteins [6]. They are expressed in the striatum and located on pre- and post-synaptic membranes [5]. The α2A-AR is expressed throughout the brain and is present in most brain areas. α2B-AR and α2C-AR show more specific distribution in the thalamus and striatum, respectively [7]. Their activation has mostly inhibitory functions on neurotransmitter, acetylcholine, and insulin release. The α2-ARs have been proposed as a therapeutic drug target for Parkinson’s disease (PD), as they have been shown to regulate dopaminergic signaling [6]. α2 adrenoceptor antagonists, such as efaroxan and idazoxan, have been tested in the quinolinic acid-induced lesion model in rats and have been described to counteract excitotoxicity by an unknown mechanism [8].

**Figure 1:**
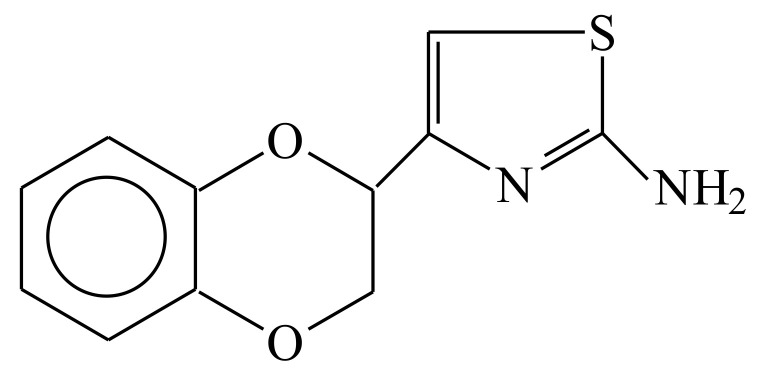
Chemical structure of 2-(2-amino-4-thiazolyl)-1,4-benzodioxane hydrochloride (beditin), a new benzodioxane derivative.

Beditin, 2-(2-amino-4-thiazolyl)-1,4-benzodioxane hydrochloride (Figure 1), is a new benzodioxane derivative that blocks vasoconstrictor reactions caused by α2-adrenergic receptor agonists and increases the body’s resistance to hypoxic–ischemic effects [9]. In experiments on animals, the drug significantly increased the rate of cerebral blood flow, lowered vascular resistance, and improved metabolic parameters in the brain during ischemia. It has also been shown that beditin leads to an increase in general and local blood flow in various parts of the brain (left and right hemispheres, brain stem, cerebellum), and increases blood flow in the heart while decreasing the tone of the coronary vessels [10]. Furthermore, beditin exhibits pronounced calcium-antagonistic properties, significantly reducing the entry of labeled calcium into the cytosol, as well as into the mitochondria and endoplasmic reticulum during ischemia [11]. A study of a methyl derivative of beditin, mesedin, on the differentiation and functional markers of neuronal precursors and astroglia led to the conclusion that mesedin can be considered as a valuable drug candidate for neurodegenerative disorders, like Alzheimer’s disease (AD), which are associated with hypoxia, beta amyloid (Aβ) accumulations, and glutamate toxicity [12]. One of the most effective and widely used α2-AR blockers among the 1,4-benzodioxane derivatives, idazoxan, contains a five-membered heterocycle with two nitrogen atoms. However, because of its high degree of toxicity, the use of idazoxan is limited to experimental studies. We sought to examine the effects of beditin in cell models of HD and show here that α2A-AR is expressed and partly elevated in cell and animal models of this disease. Further, beditin decreased cell death in ST*Hdh* cells, immortalized striatal progenitor cells, expressing 111 Q in the mouse Huntington Disease gene homologue, and ameliorated mHTT aggregation in cells overexpressing HTT exon 1. We further found that beditin treatment increased LC3-II and p62 levels in ST*Hdh* cells, as shown by an autophagic flux assay. Therefore, α2-ARs could, like in PD and AD, be interesting therapeutic targets for HD.

## 2. Results

### 2.1. α2A-Adrenergic Receptor Levels Are Increased in STHdh^Q111/Q111^ Cells

**Figure 2:**
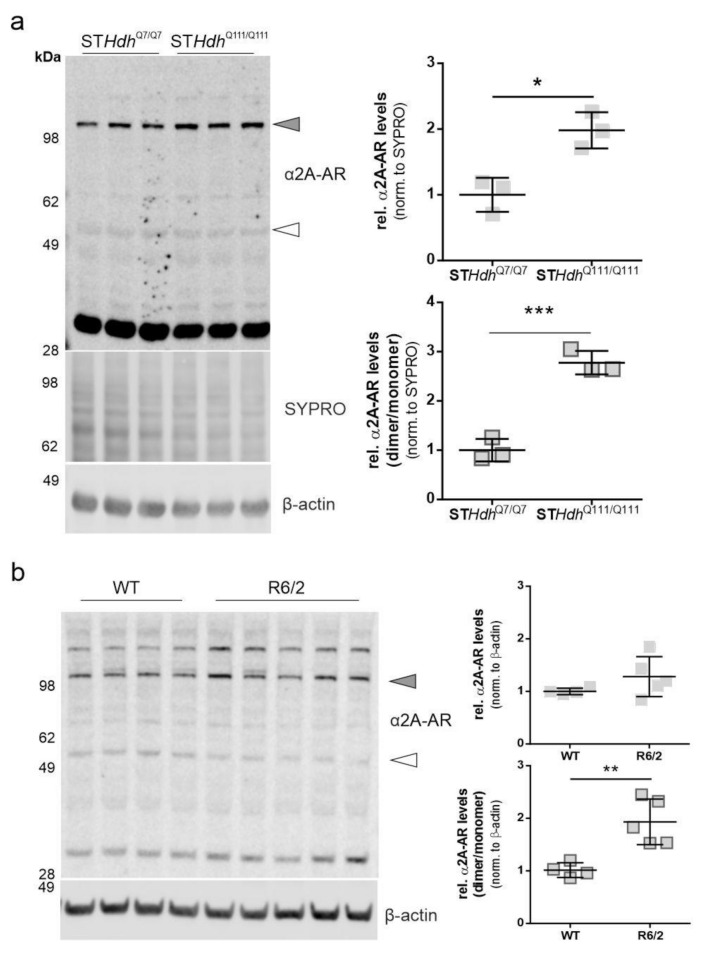
α2A- adrenoceptor (AR) levels in ST*Hdh* cells and R6/2 mice. The dimeric form of the α2A-AR is increased in ST*Hdh*^Q111/Q111^ cells and R6/2 mice. (**a**) Western blot of α2A-AR in ST*Hdh*^Q7/Q7^ and ST*Hdh*^Q111/Q111^ cells and quantification of α2A-AR signal of the band representing the dimeric form of the protein (top panel, grey shapes) and the dimer/monomer ratio (bottom panel, outlined shapes). Unpaired *t*-test with Welch’s correction. * *p =* 0.01; *n* = 3, *** *p =* 0.0007. (**b**) Western blot analysis of α2A-AR levels in striatum homogenates of 11-week-old R6/2 males vs wildtype (WT). Quantification of the α2A-AR signal of the band representing the dimeric form of the protein (top panel, grey shapes) and the monomeric form (bottom panel, outlined shapes). Unpaired *t*-test with Welch’s correction. * *p =* 0.016; *n* = 4–5; ** *p* = 0.0067; *n* = 4–5.

**Figure A1:**
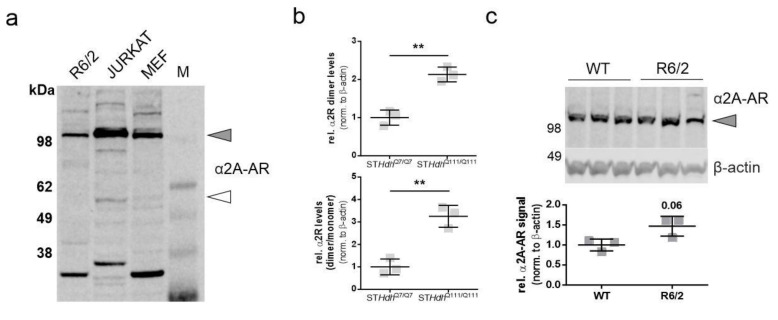
α2A-AR levels in ST*Hdh* cells and R6/2 mice. (**a**) Western blot of α2A-AR in R6/2 cortex homogenates, Jurkat homogenates, and MEF^Q111/Q111^ cells. Monomeric form indicated by unfilled arrowhead, dimeric form by grey arrowhead. (**b**) Quantification of immunoblots shown in Figure 2a with normalization to β- actin. ** *p* = 0.0021 (dimer) and ** *p* = 0.004 (dimer/monomer) (**c**) Western blot and quantification of α2A-AR levels in cortex homogenates of 11\-week-old R6/2 mice. Unpaired *t-*test with Welch’s correction *p =* 0.06, *n* = 3.

Beditin (Figure 1) was evaluated in neuronal progenitor cells (ST*Hdh* cells) derived from a knock-in mouse model [13]. ST*Hdh* cells are one of the most commonly used cell models to study HD in vitro and represent a full length HTT-expressing neuronal cell model that expresses a chimeric form of the protein at endogenous levels under mouse genomic regulatory elements. Initially, the ST*Hdh* cells were checked for the presence of the α2A-AR (Figure 2a). While the monomeric form, approximately 55 kDa in size (unfilled arrowhead), showed only a very faint band, the band corresponding to the dimeric form of the protein (>100 kDa, grey arrowhead) was increased in ST*Hdh*^Q111/Q111^ cells. To confirm the specificity of the antibody, homogenates of Jurkat cells were used, in which the antibody has been validated. Both bands have also been described in human and animal tissue [14]. Interestingly, the prominent band at approximately 32 kDa migrated distinctly in our neuronal lysates/homogenates from Jurkat cells (Figure A1a). ST*Hdh*^Q7/Q7^ and ST*Hdh*^Q111/Q111^ cells are not isogenic and differ in various characteristics, as previously described by our group [15]. Therefore, comparisons in a WT- vsHD-manner warrant precautions and confirmation in a second model. For this reason, ST*Hdh* immunoblot detections comparing both cell lines were normalized to total protein levels by SYPRO^TM^ staining, and β-actin was used as a loading control. It has been shown previously that other housekeeping proteins, such as glyceraldehyde 3-phosphate dehydrogenase (GAPDH), are dysregulated in many HD models [15,16,17]. Nevertheless, normalization via SYPRO or β-actin resulted in comparable results (Figure A1b). Unlike in the ST*Hdh* cells, the dimeric form of the α2A-AR was not significantly increased in striatum homogenates of R6/2 mice, a widely used transgenic mouse model expressing exon 1 of HTT [18], with approximately 160Q in our cohort (Figure 2b). A shift was observed in the proportion of the dimeric/monomeric form of the receptor, which showed an increased dimer to monomer ratio of the receptor in 11-week-old male R6/2 mice. This was in accordance with the findings in ST*Hdh* cells (Figure 2a). In cortex homogenates from R6/2 mice, the dimeric form showed a tendency to increase (Figure A1c, Appendix A), however in the cortex, no monomeric receptor could be detected (Figure A1a).

### 2.2. Beditin Reduces Cytotoxicity in an In Vitro Model of HD

**Figure 3:**
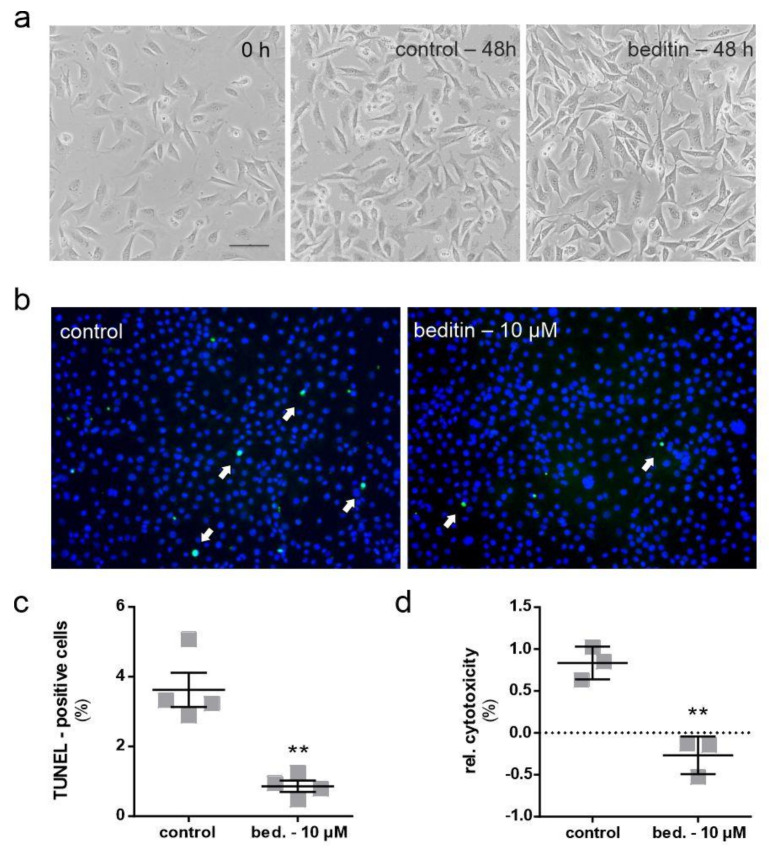
Beditin reduces cytotoxicity in ST*Hdh* cells. Cytotoxicity is reduced by treating ST*Hdh*^Q111/Q111^ cells with 10 µM of beditin. (**a**) Brightfield microscopic images of ST*Hdh*^Q111/Q111^ cells 24 h after seeding (0 h treatment) and control vs. 10 µM beditin (bed.) after 48 h of treatment. Scale bar: 20 µM. (**b**) Representative images of fluorescent terminal deoxynucleotidyl transferase dUTP nick end labeling (TUNEL) staining (white arrows) in ST*Hdh*^Q111/Q111^ cells. (**c**) Quantification of TUNEL-positive cells. Unpaired *t-*test with Welch’s correction. ** *p =* 0.0075; *n* = 4. (**d**) Lactate dehydrogenase (LDH) cytotoxicity assay in ST*Hdh*^Q111/Q111^ cells after 48 h of treatment. Unpaired *t-*test with Welch’s correction. ** *p =* 0.003; *n* = 3.

Cells treated with beditin for a prolonged time (48 h in our experiments) appeared healthy in shape and underwent proliferation, as representative images of ST*Hdh*^Q111/Q111^ cells in Figure 3a show. ST*Hdh*^Q111/Q111^ cells under standard culture conditions showed few terminal deoxynucleotidyl transferase dUTP nick end labeling (TUNEL) positive cells after a cultivation period of 48 h to 100% confluency. Nonetheless, beditin treatment decreased the number of TUNEL positive cells even further (Figure 3b,c). TUNEL labelling detects double stranded DNA breaks that occur during apoptosis. The same reduction in cytotoxicity was confirmed by the measurement of lactate dehydrogenase (LDH), which is released upon cell death into the supernatant of cells cultured in the presence of beditin compared to controls.

### 2.3. Beditin Induces Autophagy Independent of mTOR

**Figure 4:**
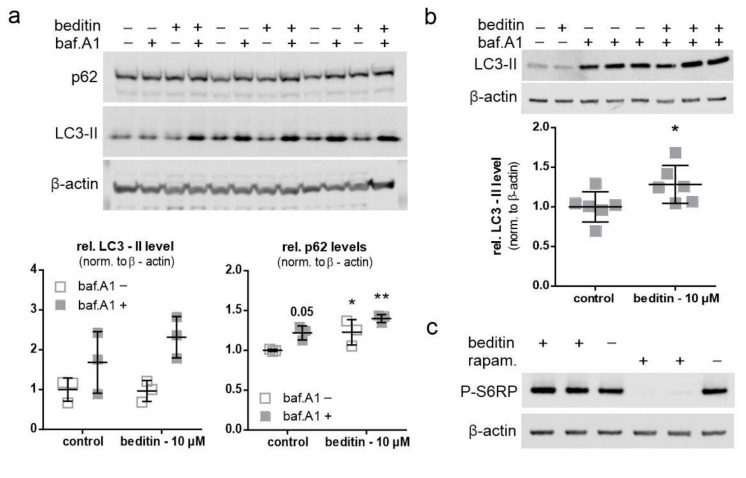
Beditin induces autophagy independent of mechanistic target of rapamycin (mTOR). Beditin induces autophagy in ST*Hdh* cells. − = control; + = indicated substance present (**a**) Western blot images of the autophagic flux assay in ST*Hdh*^Q111/Q111^ cells and respective quantification of LC3-II and p62 levels. Two-way ANOVA with Dunnett’s multiple comparison to control without bafilomycin A1 (baf.A1). p62: *p* = 0.053 (control + baf.A1), * *p* = 0.046 (beditin‒baf.A1), ** *p* = 0.002 (beditin + baf.A1), *n* = 3. (**b**) Assessment of LC3-II levels by Western blot and quantification of LC3-II levels in the presence of baf.A1. ST*Hdh* cells were treated for 4 h with beditin (10 µM). Unpaired *t*-test with Welch’s correction. * p = 0.048; *n* = 6. (**c**) Evaluation of mTOR signaling upon beditin treatment (10 µM) in comparison to rapamycin (rapam.) treatment (400 nM) by S6RP phosphorylation at Ser235/236 (P-S6RP).

**Figure A2:**
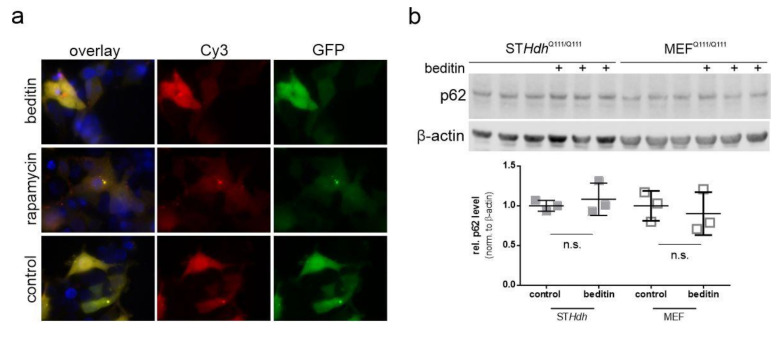
Beditin induces autophagy independent of mTOR. (**a**) Representative images of LC3B–mCherry–EGFP overexpression in HEK293T cells treated with beditin, rapamycin, or PBS (control). (**b**) Western blot and quantification of p62 levels after 24 h of beditin treatment in ST*Hdh*^Q111/Q111^ and MEF^Q111/Q111^ cells.

An autophagic flux assay was performed to evaluate the potential effects of beditin on autophagy. In the presence of bafilomycin A1 (baf.A1), a proton pump inhibitor which prevents the acidification and functionality of autophagolysosomes, autophagosomes accumulate and are protected from degradation. Therefore, in a situation when autophagy is induced, the autophagic markers microtubule-associated protein1 light chain 3 (LC3)-II and p62 accumulate in the presence of baf.A1, whereas without the addition of baf.A1, their turnover is increased. As autophagy induction is a rapid process and prolonged baf.A1 treatment causes off target effects and cytotoxicity, cells were treated for 4 h. LC3-II levels showed a moderate increase of approximately 20% in the presence of baf.A1 and beditin in comparison to controls (Figure 4a,b). Interestingly, p62 levels increased as expected in presence of baf.A1, and further increased when beditin was added, but also increased upon beditin addition alone (Figure 4a). There are different explanations for why p62 levels would accumulate. First, a late-stage block in autophagosome degradation could be caused by beditin. This seems unlikely, as the block would result in increased LC3-II levels at baseline as well. Additionally, we have visualized autophagosomes and autolysosomes using a LC3B–mCherry–EGFP reporter plasmid [19]. If functional autophagolysosomes are formed, the GFP signal is quenched in the acidic environment of the lysosomes. Therefore, if a late-stage block was induced, the amount of double positive vesicles would increase. We could not identify differences between treatments in autophagosome/autophagolysosome numbers in human embryonic kidney (HEK) 293T cells transfected with this reporter (Figure A2a), and p62 levels were no longer different when ST*Hdh*^Q111/Q111^ cells or MEF cells from the same animal model,Huntington disease knock in (HDKI) mice, were treated with beditin for 24 h (Figure A2b). Therefore, a transient upregulation of p62 might be the cause of the increased protein levels after 4 h of beditin treatment [20,21]. The mechanism by which autophagy is induced by the addition of beditin and the effects on p62 need further investigation. After 4 h of treatment, no effects on S6RP phosphorylation at Ser235/236, an mTOR-dependent phosphorylation site of this mTOR effector protein, were found. This contrasts with rapamycin (rapam.), a known mTOR inhibitor and autophagy inducer (Figure 4c), which served as a control for mTOR-dependent autophagy induction. 

### 2.4. Beditin Has Limited Effects on Soluble Full-Length Levels of Mutant Huntingtin, but Decreases HTT Fragments and HTT Exon 1 Aggregates

**Figure 5:**
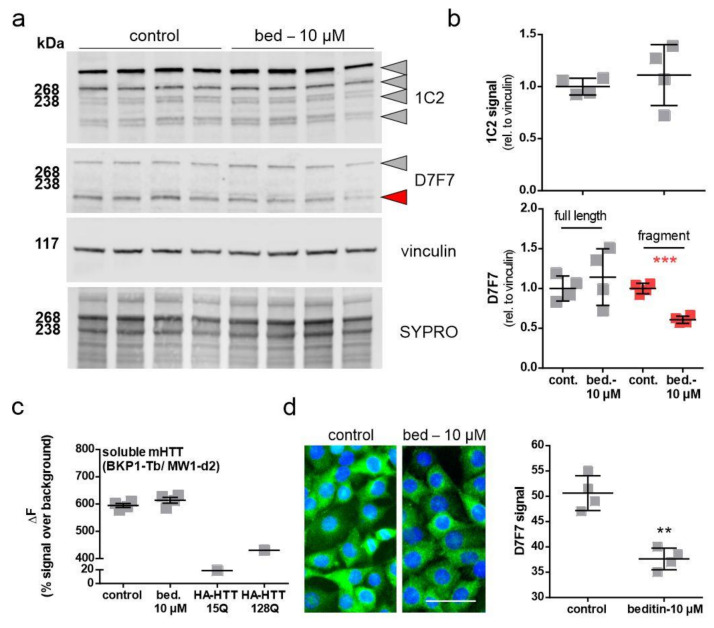
Levels of soluble mutant HTT (mHTT) in ST*Hdh*^Q111/Q111^ cells. Beditin (bed.) does not reduce total soluble mHTT levels in ST*Hdh*^Q111/Q111^ cells, treated for 48 h. (**a**) Western blot of HTT in ST*Hdh*^Q111/Q111^ cells by polyQ (1C2) and HTT (D7F7) detection and (**b**) respective quantification of total 1C2 signal (top panel, grey shapes) and D7F7 (lower panel). In the lower panel, quantification of the full-length band is shown in grey and of a lower fragment signal is shown in red. Unpaired *t*-test with Welch’s correction. *** *p* = 0.0001; *n* = 4. (**c**) time-resolved Foerster resonance energy transfer (TR-FRET) analysis of soluble mHTT levels in lysates of ST*Hdh*^Q111/Q111^ cells treated for 24 h; *n* = 4. HA-tagged HTT (HA-HTT) of different polyQ lengths serve as negative (15Q) and positive (128Q) controls for mHTT detection. (**d**) Immunocytochemistry (ICC) of mHTT (D7F7) in ST*Hdh*^Q111/Q111^ cells after 48 h of treatment and quantification of fluorescent signal; scale bar: 50 µM. Unpaired *t*-test with Welch’s correction. ** *p* = 0.0014; *n* = 4.

The effect of beditin treatment on HTT levels has been evaluated in ST*Hdh*^Q111/Q111^ cells, which only express the polyQ-expanded form of the approximately 350 kDa protein, as they derive from a knock-in model. Cells were treated for 48 h, and control vs. beditin-treated samples were analyzed by Western blot (Figure 5a,b) and TR-FRET (Figure 5c). HTT was detected by a polyQ expansion-specific antibody (1C2) and the huntingtin antibody D7F7 (Figure 5a). HTT levels measured by 1C2 immunodetection did not show differences between beditin-treated samples and controls. This was consistent for the full-length protein and for lower molecular fragments (Figure 5b, top panel). Accordingly, TR-FRET analysis did not show a difference in mHTT levels in ST*Hdh*^Q111/Q111^ cell lysates (Figure 5c). In contrast, D7F7 signal quantification revealed a lower fragment of HTT to be significantly reduced (Figure 5b, bottom panel, red squares). Moreover, immunocytochemistry (ICC) of ST*Hdh*^Q111/Q111^ cells using the D7F7 antibody showed a reduced fluorescent signal in beditin-treated samples (Figure 5d).

**Figure 6:**
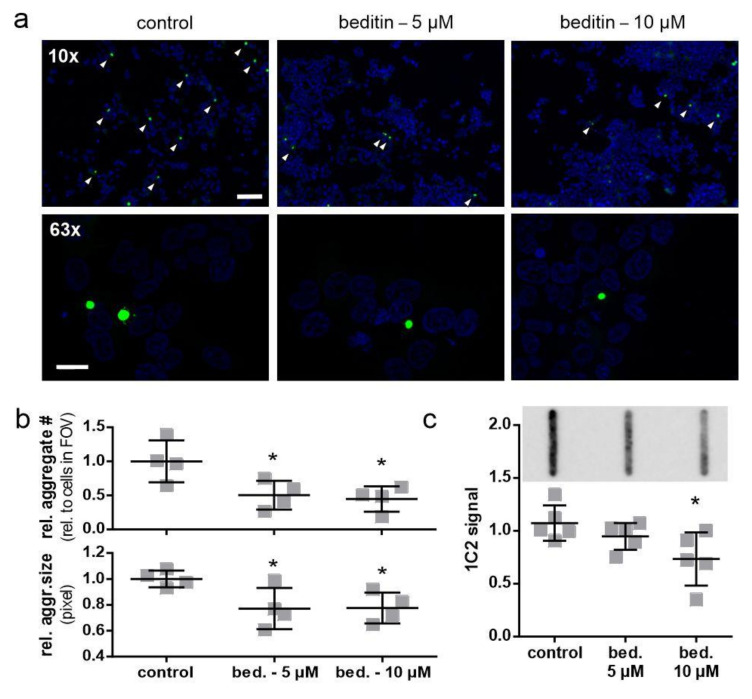
Beditin reduces mHTT exon 1 aggregation in a transient overexpression model. (**a**) Fluorescent imaging of HTT exon 1 49Q overexpression in human embryonic kidney (HEK) 293T cells for 24 h, with beditin (bed.) 5 µM and 10 µM treatment at 10× magnification (scale bar: 100 µm) for determination of aggregate count and 63× (scale bar: 20 µm) for determination of aggregate size. Aggregates at 10× magnification are highlighted by white arrow heads. (**b**) Quantification of aggregate count per cells in field of view (FOV) (top panel) and quantification of aggregate size (bottom panel). One-way ANOVA with multiple comparisons against control and Dunnett’s multiple comparison correction. Aggregate number: * *p* = 0.031 (5 µM) and * *p* = 0.018 (10 µM); *n* = 4. Aggregate size: * *p* = 0.044 (5 µM) and * *p* = 0.048 (10 µM); *n* = 4. (**c**) Filter trap analysis of HTT exon 1 49Q overexpression in HEK293T cells for 24 h with beditin 5 µM and 10 µM treatment. A representative image of filter trap immunodetection is shown above the respective quantification. One-way ANOVA with multiple comparisons against control and Dunnett’s multiple comparison correction. * *p* = 0.027 (10 µM); *n* = 5.

As we could only detect soluble levels of mHTT in ST*Hdh* cells, an aggregation assay was conducted in HEK293T cells, expressing HTT exon 1 fragments with 49Q to study the effects of beditin on the build-up of aggregates. The fast-aggregating GFP-tagged protein has the advantage that it can be directly visualized by fluorescence microscopy (Figure 6a,b) and detected by filter trap assay using a 0.45 µM cellulose acetate membrane to retain SDS-insoluble aggregates (Figure 6b). Furthermore, the repeat length of 49Q is more relevant to the pathogenic repeat lengths observed in HD patients. After 24 h, aggregate count and aggregate size were significantly reduced in the beditin-treated condition at 5 µM as well as 10 µM (Figure 6b). The filter trap assay showed a dose-dependent decline in 1C2 signal, reaching significance at 10 µM (Figure 6c).

## 3. Discussion

Our study shows that the α2A-AR is expressed in HD progenitor medium spiny neurons (MSNs), the ST*Hdh* cell line, and in the adult R6/2 mouse brain at a late disease stage and that its beditin-induced blockade in cell models of HD improves viability parameters and disease-related molecular hallmarks. The mild autophagy induction and the slowed aggregate build-up observed in beditin-treated cells argues for therapy approaches targeting this receptor in vivo.

We found a shift from the monomeric to the dimeric form of the α2A-AR in both R6/2 mice and ST*Hdh*^Q111/Q111^ cells, while dimeric levels were only tendentially elevated in R6/2 mice and monomeric levels were unchanged. Dimerization is very common for G-coupled receptors [22], and despite the denaturing conditions, re-ligated or undisrupted disulfide bonds are not uncommon. To adequately assess biological functions and possible HD-related alterations, more studies are warranted that examine the cellular localization of the receptor at different ages or disease stages and in different models. There is a limited number of studies investigating α2-ARs in HD. The gene has been mapped and found to be located in close proximity to the *HTT* gene [23]. A study in transgenic HD rats found an age-dependent decrease in α2-AR levels in the hypothalamus of female rats, associated with a disturbed circadian rhythm [24]. mRNAs of different receptors involved in neurotransmission, and the α2-AR, have been previously described to be reduced in both R6/2 and N171-82Q mice [25]. However, in peripheral blood cells of a small number of HD patients, no difference in α2-AR levels was detected [26]. Therefore, it is of great interest to further characterize the biological implications of an altered α2-AR expression pattern in different HD models.

Beditin treatment has reduced measures of cytotoxicity (TUNEL and LDH) in ST*Hdh* cells. Since TUNEL signal and LDH directly relate to DNA fragmentation during apoptosis and plasma membrane damage upon cell death, respectively, it can be assumed that beditin improves cell survival rather than acting merely on their proliferation. Future experiments should include an in vitro and in vivo assessment of cell viability and proliferation to elucidate neurogenic and neuroprotective features of beditin. α2-AR signals through GPCRs and when norepinephrine is bound, Ca^2+^ influx is triggered. This blocks the conversion of ATP to cAMP, leading to the autoinhibition of further neurotransmitter release [27]. Another aspect influencing the signaling event could be the composition and availability of GPCRs, further modulating second messengers [28]. G-protein coupled receptors readily interact to form hetero- and homodimers, whereas the sequence of events in dimerization and the effects on biological functions seem to vary between different receptors [29,30]. The α2A-AR has been shown to form homodimers and also heterodimers with the α2C-AR [31]. For all three α2-AR, it has been shown that dimers form during the transport from the ER to the plasma membrane and that α2A-AR and α2B-AR signalling is impeded by preventing transport [32]. Regarding implications for HD, it has been demonstrated that dopamine signalling can also occur through α2A-ARs, as they show similar binding affinities to dopamine as dopamine receptors [7]. This could suggest that beditin prevents dopamine-induced neurotoxicity during early phases of HD, which needs to be determined in future experiments. The heterodimer formed by the dopamine receptor D1 and the histamine H3 receptor has been recently proposed as a novel target to mitigate dopamine-induced neurotoxicity, as a H3 antagonist has been shown to prevent dopamine-mediated damages [33]. In this context, future investigations of α2A-AR and dopamine receptor heterodimerization, as well as its prevention by beditin, may shed light on the role of α2A-AR in the progression of disease pathology and provide a new strategy for therapeutic intervention in HD. GPCR signaling has a multitude of effects on cellular signaling events. Here, we found autophagy to be induced by beditin treatment, coinciding with a reduction in mHTT levels. It should be noted, however, that the increase in autophagy was mild (approximately 20%). Therefore, other mechanisms are also likely to contribute to the marked reduction in mHTT aggregation. The mechanism underlying how autophagy was induced appears to be mTOR independent, needing further dissemination of the intricate pathways that converge upon α2-AR blockade. On one hand, we can assume that an increase in cAMP upon α2-AR inhibition would lead to reduced autophagy, as cAMP (and consequently inositol trisphosphate (IP3) levels) are known negative regulators of autophagy [34]. In contrast to this, a study investigating the α2-AR agonist dexmedetomidine, showed neuroprotective effects, but also a decrease in autophagy [35]. On the other hand, the described retention of Ca^2+^ in the ER upon beditin treatment could also lead to reduced activity of Ca^2+^-regulated proteins, such as calpains. These proteases are known to act as negative regulators of autophagy, and their reduced activation could therefore lead to increased autophagy levels [36,37]. Additionally, an increased cytosolic Ca^2+^ influx leads to increased mitochondrial ATP release and therefore reduced autophagy and ultimately apoptosis [38], which is in concordance with the reduced cytotoxicity observed in this study. For this very reason, the role of autophagy as an effect of α2-AR-mediated signaling should be further investigated in a time-resolved manner. However, it should also be noted that autophagy is generally not repressed in HD. In fact, HD models have been shown to have increased levels of autophagy [39,40,41]. Other defects, like inefficient cargo loading, which leads to inefficient clearance of, e.g., protein accumulations, therefore contribute to the observed pathology [42]. Nonetheless, various studies have established that pharmacological stimulation of autophagy has beneficial effects on cell viability and huntingtin levels [39,43,44,45,46]. As encouraging evidence for a beneficial effect of α2-AR antagonists have been reported for neurodegenerative disorders of diverging origins, namely AD, PD, and now HD, a globally acting mechanism, such as autophagy, could be contributing to the positive effects observed. In PD, the drug piribedil acts as a partial dopamine D2/D3-selective agonist and blocks α2-AR. It has been proven beneficial in the treatment of motor symptoms in patients and animal models [47]. Further, a study in WT Sprague–Dawley rats has also shown neuroprotective effects of a different α2-AR antagonist, dexefaroxan [48]. In AD, the α2A-AR has been implicated in amyloidogenesis and the inhibition of α2-Ars; mesedin has been proven beneficial in cell models [12] and a mouse model of AD [33,49], while levels of α2-AR have been found to be reduced in AD patients [50]. Ultimately, it will be interesting to further investigate how beditin influences intracellular α2-AR-mediated signaling events and whether it can prevent cellular damage in vivo in HD.

## 4. Materials and Methods

### 4.1. Cell Culture and Treatment

ST*Hdh* cell lines (ST*Hdh*^Q7/Q7^ and ST*Hdh*^Q111/Q111^) [13], immortalized cells derived from murine striatal primordia, were obtained from Coriell Cell Repositories (Coriell Institute for Medical Research; Camden, NJ, USA). The two progenitor cell lines either contain 7 CAGs in the wild-type mouse gene locus or 111 within the chimeric exon 1 of *HTT*, homozygously knocked into the mouse locus [51]. Cell passages 4–12 were used for the experiments. All cell lines were maintained in Dulbecco’s modified Eagle’s medium (DMEM, Gibco, Thermo Fisher, Waltham, MA, USA), supplemented with 10% fetal bovine serum and 1% penicillin/streptomycin at 37 °C in 5% CO_2_. The generation of mouse embryonic fibroblasts (MEF) cells has previously been described [15]. The medium for ST*Hdh* cells was further complemented with 1% geneticin (G418, A2912, Biochrome, Berlin, Germany). All cell lines routinely tested negative for mycoplasma contamination by PCR. Cells were seeded out one day before treatment. At approximately 80% confluency, STHdh cells were treated with beditin (10 μM beditin dissolved in distilled water). Bafilomycin A1 (baf.A1; InvivoGen, San Diego, CA, USA) was added at 50 nM for autophagic flux assays.

For immunocytochemical analyses, ST*Hdh* cells were plated on coverslips in 6 cm dishes (500,000 cells per dish) and cultured in high glucose GlutaMAX Dulbecco’s modified Eagle’s medium (DMEM, Gibco, Thermo Fisher, Waltham, MA, USA) with 10% fetal bovine serum, 1% penicillin/streptomycin and 1% pyruvate in humidified atmosphere of 5% CO_2_ at 37 °C. Then, 24 h after seeding, the medium was exchanged and the cells were incubated for 48 h with or without beditin. Beditin (2-(2-amino-4-thiazolyl)-1,4-benzodioxane hydrochloride) was synthetized and kindly provided by S. Vardanyan, L.A. Mnjoyan Institute of Fine Organic Chemistry, Yerevan, Armenia.

### 4.2. Cell Transfection and Protein Overexpression

Briefly, constructs for the expression of a HTT exon 1 fragment with 20Q or 49Q were generated by inserting the respective DNA fragments into the enhanced Green Fluorescent Protein (EGFP) pEGFP-N1 vector (Clontech Laboratories, Mountain View, CA, USA) via the XhoI and HindIII restriction sites. HEK293T cell transfection with HTT–Exon1–eGFP–20Q/49Q or pBABE–LC3B–mCherry–EGFP [19] was performed with TurboFectin 8.0 (OriGene Technologies, Inc., Rockville, MD, USA) according to the manufacturer’s protocol for filter trap analysis. pBABE–puro mCherry–EGFP–LC3B was a gift from Jayanta Debnath [52].

### 4.3. Immunocytochemistry

After 48 h incubation with beditin, the cells (*n* = 4 per treatment condition) were washed with phosphate buffered saline (PBS), fixed with 4% formaldehyde for 10 min at room temperature (RT), and again washed with PBS for 10 min. Incubation with rabbit monoclonal anti-HTT (D7F7, 1:800, Cell Signaling, Danvers, MA, USA) primary antibodies was performed at room temperature (RT) for 1 h. For TUNEL staining, the In Situ Cell Death Detection Kit, Fluorescein (Cat.# 11684795910, Merck, Darmstadt, Germany) was used according to the manufacturer’s protocol. After washing twice with PBS, the cells were incubated in the dark for 1 h at RT with fluorescein isothiocyanate (FITC)-conjugated goat anti-rabbit IgG (1:500) from Dianova (Jackson Immunoresearch, West Grove, PA, USA). The cells were then washed with PBS containing 0.05% Triton X-100 (Sigma-Aldrich, St. Louis, MO, USA), mounted with Vectashield Mounting Medium containing 4′,6-diamidine-2′-phenylindole dihydrochloride (DAPI, Vector Laboratories, Burlingame, CA, USA), and evaluated by fluorescence microscopy with an Olympus BX51 and analySIS Software (Olympus, Hamburg, Germany). Quantification of TUNEL and HTT-positive cells was performed from 8–9 images per cell treatment condition.

### 4.4. Western Blot and Immunodetection

Analysis of autophagy markers was performed as previously described [46]. Cells were disrupted by vortexing in radioimmunoprecipitation assay (RIPA) buffer (50 mM Tris-HCl pH 7.5, 150 mM NaCl, 1% NP-40, 0.5% sodium deoxycholate, 0.1% SDS) containing protease inhibitors (cOmplete™, Roche, Basel, Switzerland) and a phosphatase inhibitor (phosSTOP, Roche, Basel, Switzerland). Cell and brain homogenates were incubated for 30 min on ice and vortexed every 10 min. Lysates were obtained after centrifugation for 20 min, 16,100 × *g* at 4 °C. Protein concentration was determined by Bradford assay (Bradford reagent, Bio-Rad Laboratories, Inc., Hercules, CA, USA) spectroscopically and adjusted to 30 µg total protein per sample. Proteins were separated and blotted using standard protocols. Bolt™ 4–12% Bis-Tris Plus gels (Thermo Fisher, Waltham, MA, USA) were used with 2-(N-morpholino)ethanesulfonic acid (MES) running buffer (50 mM MES, 50 mM Tris, 0.1% SDS, 1 mM ethylenediaminetetraacetic acid (EDTA)). All proteins, except for LC3B, were transferred onto a 0.2 µm nitrocellulose membrane (Amersham Protran^TM^ Premium, GE Healthcare, Little Chalfont, UK). LC3B was blotted onto a 0.2 µm polyvinylidene fluoride (PVDF) membrane (Amersham Hybond P, GE Healthcare, Little Chalfont, UK). The primary antibodies used were: α2A-AR (1:750, SAB4500548, Sigma-Aldrich, St. Louis, MO, USA), LC3 (1:200, clone 5F10, NanoTools, Teningen, Germany), p62 (1:1000, #5114, Cell Signaling, Danvers, MA, USA), P-S6 ribosomal protein (Ser235/236) (1:1000, #2211, Cell Signaling, Danvers, MA, USA), anti-polyglutamine-expansion diseases marker antibody (1:2000, clone 5TF1-1C2, MAB1574, Merck KGaA, Darmstadt, Germany), huntingtin (1:1000, clone D7F7, Cat.#5656, Cell Signaling, Danvers, MA, USA), β-actin (1:20,000, clone AC-15, A5441, Sigma-Aldrich, St. Louis, MO, USA), and vinculin (1:1000; clone E1E9V, Cat.#13901, Cell Signaling; Danvers, MA, USA). For detection, respective secondary IRDye^®^ antibodies (goat anti-mouse 680LT, goat anti-mouse 800CW and goat anti-rabbit 800CW (1:10,000, LI-COR Biosciences, Bad Homburg, Germany)) were incubated for 1 h and detected with a LI-COR Odyssey^®^ Fc (LI-COR Biosciences, Bad Homburg, Germany). Quantification of the signal was performed with LI-COR Image Studio Lite software, version 4.0.21, and normalized to the β-actin signal, unless stated differently. Full immunodetections are shown in Supplementary Materials. 

### 4.5. TR-FRET

Huntingtin measurements by time-resolved Foerster resonance energy transfer (TR-FRET) were performed as previously described [53,54] with the following modifications: soluble mutant huntingtin was detected by a combination of the N-terminally binding monoclonal anti-HTT antibody BKP1 [55] labeled with the Tb (donor) fluorophore and the anti-polyglutamine antibody MW1 [56] labeled with the d2 (acceptor) fluorophore (labeling by CisBio, Codolet, France). Briefly, lysates of beditin-treated ST*Hdh* cells (lysed in 300 mM NaCl, 50 mM Tris, 2 mM MgCl_2_, 0.05% NP40, containing protease inhibitors (cOmplete™, Roche, Basel, Switzerland) and frozen at −80 °C were diluted in tris-buffered saline with TBST (25 mM Tris pH 7.4, 137 mM NaCl, 2.7 mM KCl, 3% (*v*/*v*) Tween-20) to a final concentration of 1 µg/µL, and 5 µL of the diluted sample was transferred to a low-volume white ProxiPlate 384 TC Plus plate (PerkinElmer, Waltham, MA, USA). Transfected HEK293T cells expressing pCI-neo HA-HTT 15Q/128Q vectors (kindly provided by Michael Hayden (CMMT, Vancouver, Canada)) were lysed in RIPA buffer. Next, 1 µL detection buffer (50 mM NaH_2_PO_4_ pH 7.4, 400 mM NaF, 0.1% BSA, 0.05% Tween-20) containing the TR-FRET antibody mix (0.3 ng BKP1-Tb + 3 ng MW1-d2) was added to the sample and incubated at 4 °C for 22 h. After equilibration to RT for 15 min, signals were detected at 620 nm and 665 nm using an EnVision Multimode Plate Reader with a TRF-laser unit (PerkinElmer, Waltham, MA, USA).

### 4.6. LDH Assay

Effects on cytotoxicity were determined using the Cytotoxicity Detection Kit (LDH) (Roche, Basel, Switzerland). The procedure was performed according to the manufacturer’s protocol. In brief, cells were seeded at 10,000 per well in 96-well plates and incubated for approximately 12 h before treatment and a further incubation for 48 h. Absorption was measured with MWGt Synergy HT plate reader (BioTek Instruments, Winooski, VT, USA) and analyzed with Gen5 2.01 software (BioTek Instruments, Winooski, VT, USA).

### 4.7. Filter Trap and Microscopic Aggregate Analysis

HEK293T cells were transiently transfected with the eGFP–HTT–Exon1–20Q/49Q constructs. Total expression time was 72 h; treatment with beditin started 24 h before harvest. Cells were detached with ice-cold Dulbecco’s phosphate-buffered saline (DPBS, Thermo Fisher, Waltham, MA, USA) and lysed in RIPA buffer (see Western blot); homogenates were prepared by sonication (Bandelin Sonopuls HD2070, BANDELIN electronic GmbH & Co. KG, Berlin, Germany). For the detection of aggregates, a 0.2 µm cellulose acetate membrane (OE66, Sigma-Aldrich, Merck KGaA, Darmstadt, Germany) was preincubated in 2% SDS in DPBS for 5 min and 12.5 µg of protein was applied in a Minifold^®^ II Slot Blot System (Schleicher & Schuell, GmbH, Erdmannhausen, Germany). Membranes were washed in TBS and blocked in 5% skim milk powder in TBS.

For analysis of aggregate number and size, transfected HEK293T cells on coverslips were treated for 24 h before fixation with 4% paraformaldehyde in DPBS for 15 min at RT. After washing with DPBS, coverslips were mounted onto slides with Vectashield Mounting Medium containing 4′,6-diamidine-2′-phenylindole dihydrochloride (DAPI, Vector Laboratories, Burlingame, CA, USA) and evaluated by fluorescence microscopy. Images were acquired using a Zeiss Axioplan microscope (10×/0.75 objective, AxioCam MRc for aggregate count and 63× with an Apotome for aggregate size) (Zeiss, Jena, Germany). Transfection rates were comparable between treatments. Aggregates were identified by particle analysis (ImageJ; [57]) and normalized to cell count (DAPI signal). Cells and aggregates per cell were counted on 8 pseudo-randomized pictures per condition and in 4 independent experiments. For aggregate size analysis, 30 aggregates per condition were analyzed by measuring the area with ImageJ in 4 independent experiments.

### 4.8. Statistical Analysis

All data are presented as individual measurements (square shapes) with mean and standard deviation (SD) or standard error of the mean (SEM). Statistical analysis was performed with GraphPad Prism 6.00 for Windows (GraphPad Software, Inc; San Diego, CA, USA). Comparison of two groups was performed with unpaired, two-tailed *t*-test. Comparison of multiple treatments was performed by one-way/two-way ANOVA, with multiple comparison correction (Dunnett’s). Significance level, α, was set to 0.05.

## 5. Conclusions

Studies in other neurodegenerative disorders have pointed at the α2-AR as a new therapeutic target to ameliorate cellular demise and protein deposition. Therefore, we have explored the effects of beditin, an α2-AR antagonist, in cell models of HD. We show here that beditin exerts neuroprotective effects and reduces the build-up of aggregates *in vitro*. Hence, our study identifies the α2-AR as a therapeutic target in HD and suggests beditin as a potential candidate for therapeutic intervention in HD.

## Data Availability

Data can be made available from the corresponding author upon reasonable request.

## Supplementary Materials

The following are available online at https://www.mdpi.com/1424-8247/14/3/257/s1, Full immunodetections.
