# Supplementary material for: The Novel Alpha-2 Adrenoceptor Inhibitor Beditin Reduces Cytotoxicity and Huntingtin Aggregates in Cell Models of Huntington’s Disease
Source: Pharmaceuticals (Basel). 2021 Mar 12;14(3):257. doi: 10.3390/ph14030257 (PMC7998230; doi:10.3390/ph14030257)

Novel alpha-2 adrenoceptor inhibitor beditin reduces cytotoxicity and huntingtin aggregates in cell models of Huntington disease

Elisabeth Singer[1,2,6#], Lilit Hunanyan[3#], Magda M. Melkonyan[3], Jonasz J. Weber[1,2,6], Lusine Danielyan[4, 5] and Huu Phuc Nguyen[6]

- full immunodetections -

Figure 2 a)

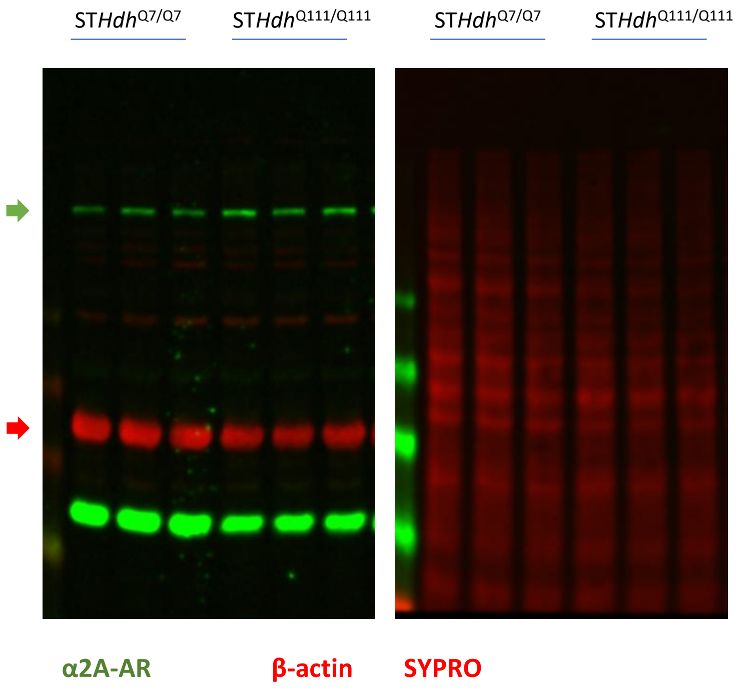

Figure 2 b)

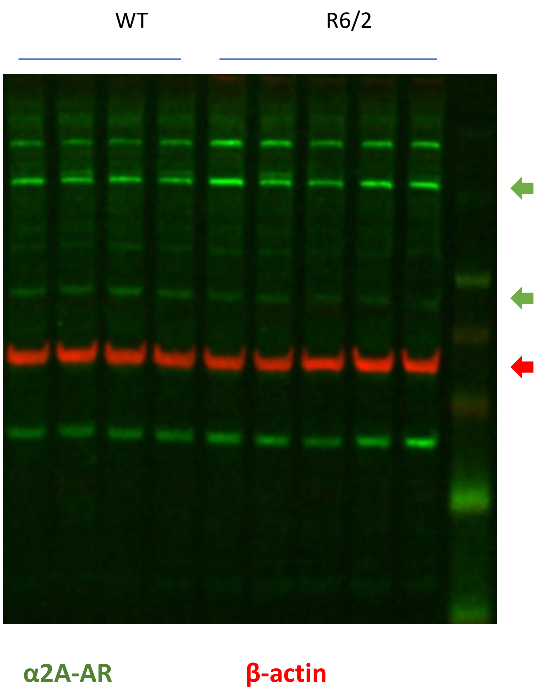

Figure 4 a)

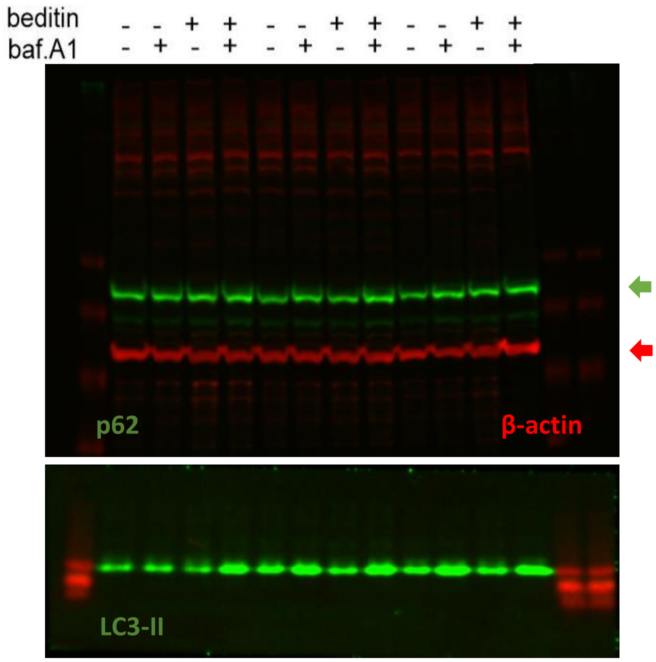

Figure 4 b)

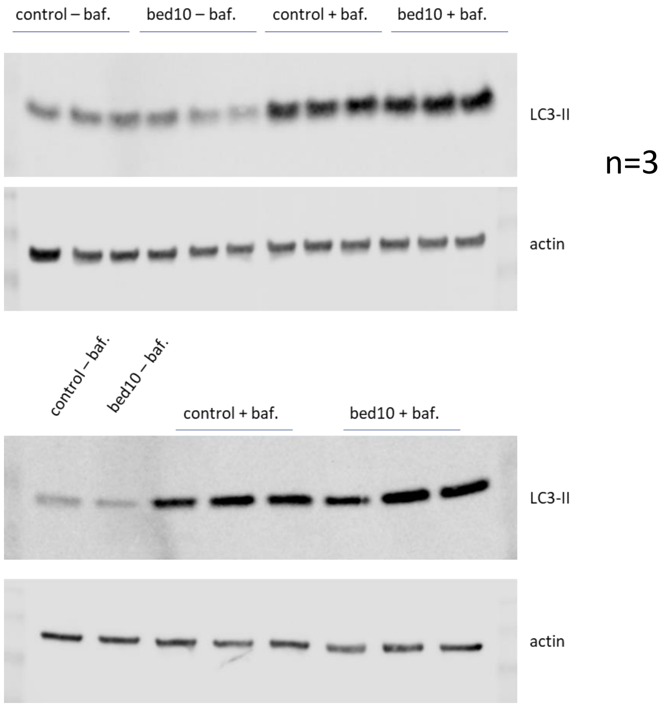

n=3, shown in Figure 4b)

Figure 5 a)

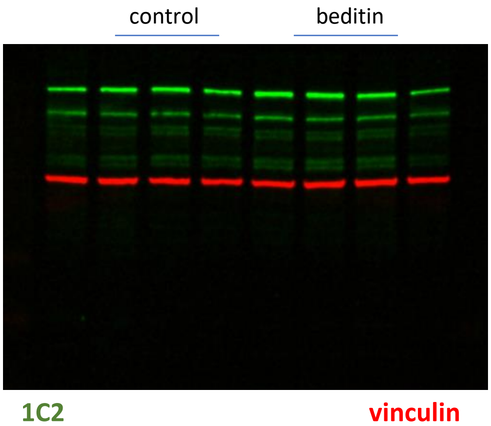

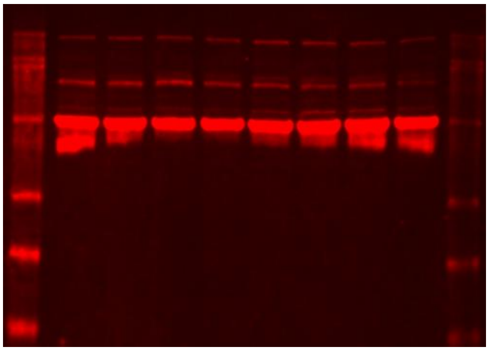

D7F7/ vinculin (blotted on the same membrane, sequentially)

Figure 6 c)

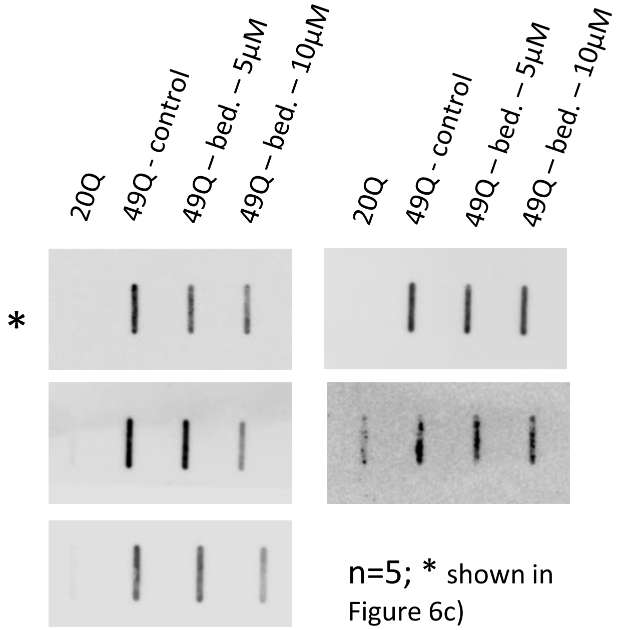

n=5; * shown in Figure 6c)

Supplementary Figure 1 b):

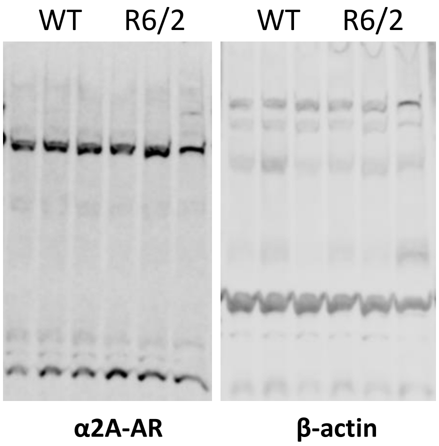

Supplementary Figure 2:

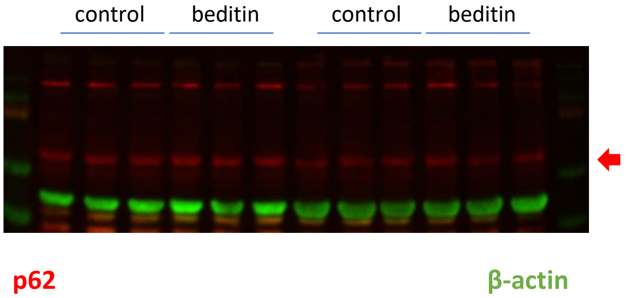

Supplement: Supplementary file 1 [file pharmaceuticals-14-00257-s001.pdf]
